# An Electrochemical Quartz Crystal Microbalance Multisensor System Based on Phthalocyanine Nanostructured Films: Discrimination of Musts

**DOI:** 10.3390/s151129233

**Published:** 2015-11-19

**Authors:** Celia Garcia-Hernandez, Cristina Medina-Plaza, Cristina Garcia-Cabezon, Fernando Martin-Pedrosa, Isabel del Valle, Jose Antonio de Saja, Maria Luz Rodríguez-Méndez

**Affiliations:** 1 Department of Inorganic Chemistry, Engineers School, University of Valladolid, Valladolid 47011, Spain; E-Mails: Celiagarciahernandez@gmail.com (C.G.-H.); Crismedina@eii.uva.es (C.M.-P.); 2 Department of Materials Science, Engineers School, University of Valladolid, Valladolid 47011, Spain; E-Mails: crigar@eii.uva.es (C.G.-C.); fmp@eii.uva.es (F.M.-P.); 3 Department of Electronic Technology, Engineers School, University of Valladolid, Valladolid 47011, Spain; E-Mail: isaval@eii.uva.es; 4 Department of Condensed Matter Physics, Faculty of Sciences, University of Valladolid, Valladolid 47011, Spain; E-Mail: sajasaez@fmc.uva.es

**Keywords:** LbL, EQCM, phthalocyanine, must, grapes, electronic tongue

## Abstract

An array of electrochemical quartz crystal electrodes (EQCM) modified with nanostructured films based on phthalocyanines was developed and used to discriminate musts prepared from different varieties of grapes. Nanostructured films of iron, nickel and copper phthalocyanines were deposited on Pt/quartz crystals through the Layer by Layer technique by alternating layers of the corresponding phthalocyanine and poly-allylamine hydrochloride. Simultaneous electrochemical and mass measurements were used to study the mass changes accompanying the oxidation of electroactive species present in must samples obtained from six Spanish varieties of grapes (Juan García, Prieto Picudo, Mencía Regadío, Cabernet Sauvignon, Garnacha and Tempranillo). The mass and voltammetric outputs were processed using three-way models. Parallel Factor Analysis (PARAFAC) was successfully used to discriminate the must samples according to their variety. Multi-way partial least squares (N-PLS) evidenced the correlations existing between the voltammetric data and the polyphenolic content measured by chemical methods. Similarly, N-PLS showed a correlation between mass outputs and parameters related to the sugar content. These results demonstrated that electronic tongues based on arrays of EQCM sensors can offer advantages over arrays of mass or voltammetric sensors used separately.

## 1. Introduction

The combination of an array of partially selective sensors with overlapping specificities with advanced mathematical signal-processing methods has yielded a new sensing technology for chemical analysis in liquid media, the so called electronic tongue (ET) [1,2]. ETs are holistic systems that provide global and qualitative information about samples instead of quantitative data about specific compounds. However, if the data matrix obtained by such multisensor systems is analyzed with adequate chemometric processing tools, descriptive or predictive information of particular parameters can be extracted [3,4].

Arrays of sensors dedicated to the analysis of liquids are usually based on electrochemical sensors including potentiometric [5], amperometric [6], voltammetric [7,8,9] or impedimetric ones [10]. Many efforts have been dedicated to the discrimination of wines and musts using electronic tongues [2,3,8,9,11,12,13,14]. Voltammetric electrodes chemically modified with electrocatalytic materials are particularly suitable for the analysis of wines because voltammograms contain information related to the redox and ionic species present in wines [15]. For this reason, e-tongues based on voltammetry have been able to discriminate red wines with different antioxidant capability [16], wines elaborated with different varieties of grapes [17], to detect adulterations [8] or to follow the ageing of red wines using different methods [18].

The intrinsic complexity, richness and cross-selectivity of the signals generated by voltammetric sensor arrays are an advantage because the datasets contain meaningful information about the samples. Using partial least squares regression analysis (PLS), correlations could be established between the voltammetric curves (not only specific peaks) and the polyphenol content, or the acidity of red wines [12,16,19]. Finally, multi-transduction systems are more and more popular, since they combine different classes of sensors that provide complementary information [13,20,21,22,23,24].

Quartz Crystal Microbalances (QCM) are a different approach where the sensing element is a coated resonator. The interaction with the sample results in a change in the mass of the crystal which affects the frequency at which the crystal oscillates. QCM sensors modified with a variety of materials (porphyrins, CNTs, calixarenes and biomolecules, among others) have been widely used in electronic noses to analyze vapors with sub-nanogram sensitivity [25,26,27,28]. QCMs can also work in an aqueous environment and they have been able to detect a variety of substances in solution [29,30]. However, only few examples of electronic tongues based on QCM sensors for the analysis of liquids have been reported [31,32,33].

There are numerous methods to functionalize QCM surfaces. They include hydrophobic bonds, ionic bonds, hydrogen bonds or electrostatic interactions, among others [34]. Nanostructured films show enhanced surface to volume ratios that can increase the sensitivity of the sensors [35]. In fact, QCM sensors have been prepared using Langmuir-Blodgett (LB) [36], Layer by Layer (LbL) [37], Self-Assembling Monolayer (SAM) [38], Electrodeposition (EDP) [39,40], Plasma-Polymerized Films (PPF) [41] or Molecular Imprint (MIP) [42] techniques.

Apart from their sub-nanogram sensitivity, another possible advantage of QCM sensors for the analysis of liquids, is that the same device can be used to obtain simultaneously mass changes and electrochemical signals. For this reason, the Electrochemical Quartz Crystal Microbalance (EQCM) has become a well-established technique for the investigation of mass changes associated with electrochemical surface processes such as adsorption of enzymes [43], electrodeposition [44], electropolymerization [45], ion insertion [46] or redox processes [33]. In this way, EQCM can provide higher amounts of information about a sample than electrochemistry or mass measurements performed separately [33,47,48].

EQCM sensors covered with phthalocyanines have been successfully used to analyze phenols present in wines [33]. The reason for their success is that phthalocyanines show electrocatalytic properties towards a variety of analytes including phenols [33,49,50]. In addition, nanostructured electrodes based on phthalocyanines can be prepared by electrodeposition [40], the Langmuir-Blodgett (LB) [35,51] or the Layer by Layer (LbL) technique [52,53,54] on different substrates such as indium tin oxide (ITO) or platinum.

The objective of this work was to combine EQCM sensors to form an array able to discriminate musts with different phenolic content, obtained from different varieties of Spanish grapes. For this purpose, QCM substrates were modified with three different metallophthalocyanines using the LbL technique. The response of the array of sensors towards the must samples was studied by recording cyclic voltammetry and mass changes synchronously. The capability of the system to discriminate grapes of different varieties was evaluated by means of parallel factor analysis (PARAFAC). The relationship between the voltammetric or the mass signals with the results obtained by chemical analysis was studied by means of multi-way partial least squares (N-PLS). The complementarity of the information provided by the electrochemical and mass outputs was discussed.

## 2. Experimental Section

### 2.1. Chemicals and Grape Samples

All chemicals and solvents were purchased from Sigma-Aldrich (St. Louis, MO, USA) and used without further purification. The solutions were obtained by dissolving substances in deionized water (resistivity of 18.2 MΩ·cm^−1^) acquired from a Milli-Q system (Millipore, Billerica, MA, USA).

Three water soluble tetrasulfonate acid metallophthalocyanines (MPc^SO3^) were used as the anions in the LbL films. They included iron(III) phthalocyanine tetrasulfonic acid monosodium salt, copper(II) phthalocyanine tetrasulfonic acid tetrasodium salt, and nickel(II) phthalocyanine tetrasulfonic acid tetrasodium salt; (denoted as FePc^SO3^, NiPc^SO3^ and CuPc^SO3^ respectively). Polyallylamine hydrochloride (PAH), was used as the polycation in LbL films.

Six varieties of red grapes were included in the study (Juan García, Tempranillo, Prieto Picudo, Mencía Regadío, Cabernet, Garnacha). They were harvested in September 2013 from the vineyards “Bodega Cooperativa de Cigales” and “Instituto Tecnológico Agrario de Castilla y León (ITACYL)”, both located in the Valladolid area of Castilla y León in Spain. To obtain the musts, 200 berries were introduced in a plastic bag and crushed for one minute. The Oenological Centre of Castilla y León carried out the chemical analysis following international regulations [55]. Parameters analyzed included the typical indicators of the glucose content: sugar content (g/L), degree 16.8 and Brix degree. The classical indicator of the polyphenolic content, the Total Polyphenol Index (TPI) was also analyzed. The polyphenolic content was also evaluated using the Folin-Ciocalteau method. The results are collected in Table 1.

**Table 1:** Results of the chemical analysis carried out by traditional chemical methods.

Grape Variety	Sugar Content (g/L)	Brix Degree	Total Polyphenol Index. TPI	Degree 16.8	Polyphenol Content. Folin-Ciocalteau Method (g/L)
Prieto Picudo	224.1	22.89	19	13.31	0.46
Garnacha	187.4	19.68	15	11.13	0.38
Cabernet-Sauvignon	246.4	24.75	28	14.64	0.62
Tempranillo	209.1	21.53	28	12.42	0.52
Juan García	216.0	22.18	29	12.83	0.69
Mencía Regadio	203.3	21.05	23	12.08	0.54

### 2.2. Electrochemical Measurements

Electrochemical quartz crystal microbalance experiments were carried out with a quartz crystal microbalance (QCM200 + QCM25 Crystal Oscillator, purchased from Standford Research Systems, Sunnyvale, CA, USA) connected to a Parstat 2273 potentiostat/galvanostat (EG&G, Oak Ridge, TN, USA). The mass-sensitive oscillators were 2.54 cm diameter, 5 MHz AT-cut planoconvex quartz crystals coated with platinum.

The oscillators were used simultaneously to register mass changes and as the working electrode of the electrochemical measurements. PC software displays the relative frequency changes in synchronicity with the electrochemical data.

Cyclic voltammetry was carried out using a conventional three-electrode cell. The reference electrode was Ag|AgCl/KCl 3 mol·L^−1^ and the counter electrode was a platinum sheet with a surface of 1 cm^2^. Cyclic voltammograms were registered at a sweep rate of 0.1 V·s^−1^. The variations of mass taking place simultaneously to the cyclic voltammetry experiment were registered. All the samples were measured seven times with each sensor to check the robustness of the experiments.

### 2.3. Calibration of the Quartz Crystal

A calibration of the quartz crystal was carried out to determine the sensitivity factor (also called Sauerbrey constant) by galvanostatic deposition of copper on the platinum surface. Sauerbrey constants have been used to calculate mass shifts during the experiment (Equation (1)):

(1) $$∆f=-C_{f}\cdot ∆m$$

where ∆*f* is the observed frequency change (Hz), *C_f_* is the Sauerbrey constant (56.6 Hz·cm^2^·µg^−1^ for a 5 MHz At-cut quartz crystal at room temperature) and ∆*m* is the change in mass per unit area (µg·cm^−2^).

A 0.5 mol·L^−1^ solution of CuSO_4_ in 0.1 mol·L^−1^ H_2_SO_4_ (pH = 1.37) was used to deposit copper on the platinum electrode using chronopotentiometry (−2 mA, 70 s). The plot of the frequency shift *vs*. charge showed excellent linearity (*y* = 7 × 10^−5^*x* − 0.0632; *R*^2^ = 0.9996). According to the linear plot obtained in the calibration process, the Sauerbrey constant of the quartz crystal obtained was 59.4 Hz·cm^2^·µg^−1^, which is consistent with the theoretical value of 56.6 Hz·cm^2^·µg^−1^.

### 2.4. Sensor Preparation

The platinum-coated quartz crystal substrates were cleaned using a mixture of H_2_SO_4_ and H_2_O_2_ (piranha solution; 3:1 mixture of sulfuric acid and 30% hydrogen peroxide) and thoroughly rinsed in deionized water (resistivity of 18.2 MΩ·cm^−1^) before use.

The LbL films were grown using PAH as the positive layer and iron, nickel or copper tetrasulfonate phthalocyanines (FePc^SO3^, NiPc^SO3^ or CuPc^SO3^) as the negative layer using a previously published procedure [56]. Water solutions of PAH (0.5 g·L^−1^) and of the corresponding phthalocyanine (0.05 g·L^−1^) were used to build the films. LbL films were fabricated by successive immersions of the QCM substrate in the PAH and phthalocyanine solutions. The following sequence of immersions was followed: (1) PAH solution (5 min); (2) deionized water gently stirred to remove excess of non-adsorbed PAH (1 min); (3) MPc^SO3^ solution (5 min); (4) deionized water gently stirred to remove excess and non-adsorbed MPc^SO3^ (1 min). After these four steps a bilayer was formed and more layers are grown by repeating the sequence. After each electrochemical measurement, QCM substrates were cleaned by immersing the Pt/quartz device in HNO_3_ 60% (sonication during 1 min) to remove LbL films. QCM devices were then rinsed in deionized water and dried with nitrogen gas.

### 2.5. UV-Visible Characterization

The growth of LbL films was monitored with UV-Vis absorption spectroscopy (UV-2600 model, purchased from Shimadzu, Kyoto, Japan) by testing the linearity between the number of layers deposited and the absorbance.

### 2.6. Multisensor System Statistical Analysis

A multisensor system was constructed using the signals obtained from four sensors: the already described FePc^SO3^/PAH, NiPc^SO3^/PAH and CuPc^SO3^/PAH LbL films and a bare Pt quartz crystal microbalance. The multivariate data analysis was performed by using Matlab v2014b (The Mathworks Inc., Natick, MA, USA) and The Unscrambler (CAMO Software AS, Oslo, Norway). Voltammograms and massograms were pre-processed by using an adaptation of a data reduction technique based on predefined response “bell-shaped-windowing” curves called “kernels” [15,57]. Using this method, 10 variables were obtained from each voltammogram and from each massogram. These data were used as the input variable in statistical analysis. A non-supervised multivariate method, the Parallel Factor Analysis (PARAFAC) was used to analyze the curves and to evaluate the capability of discrimination of the array of sensors. Multi-way partial least squares (N-PLS) was used to evidence the correlations between the voltammetric and mass outputs and the chemical indicators.

## 3. Results and Discussion

### 3.1. UV-Visible characterization

LbL films were prepared using the four-step sequence described above. The procedure was repeated 20 times to obtain 20 bilayer-thick films. The growth of bilayers was monitored every four bilayers by UV-vis absorption spectroscopy. The characteristic B band at shorter wavelengths and Q band at longer wavelengths, which are attributed to HOMO→LUMO electronic transitions of the π electrons of the Pc ring, could be clearly seen. The features observed in the UV-Vis absorption spectra are assigned to the MPc^SO3^ since PAH does not absorb within this wavelength range.

**Figure 1:**
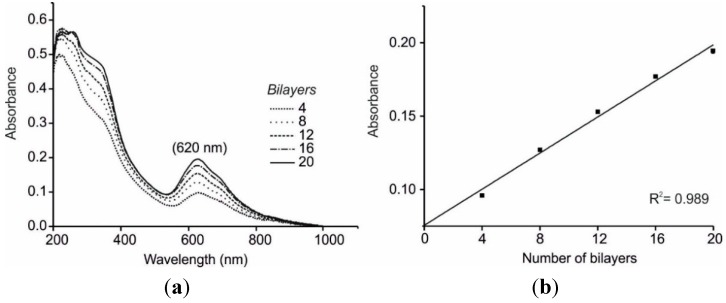
UV-Vis characterization of 4–20 CuPc^SO3^/PAH LbL bilayers. (**a**) UV-Vis absorption spectra; (**b**) Linear correlation between absorbance *vs*. number of bilayers.

The absorbance of the Q band increased linearly with the number of bilayers. This is illustrated in Figure 1 for the CuPc^SO3^/PAH film where the absorbance at 620 nm *vs.* the number of deposited bilayers is represented, confirming the good quality of the deposition. This shows that a similar amount of material is transferred onto the substrate per deposited layer confirming a uniform growth of the LbL films. The quality of the layers (and hence the linearity regression coefficient) decreased when more than 20 bilayers were deposited. For this reason, further studies were carried out with 20 bilayers.

Similar results were obtained with the three phthalocyanines tested and the only difference was the value of the Q band position (640 nm for FePc^SO3^/PAH, 620 nm for NiPc^SO3^/PAH and 620 for CuPc^SO3^/PAH), which are in good agreement with previous results [53,54].

In addition, the absorbance values registered increased when advancing in the transition metal series (FePc^SO3^/PAH < NiPc^SO3^/PAH < CuPc^SO3^/PAH films) (for instance, the values of Q band absorbance registered for 20 bilayers were FePc^SO3^/PAH: 0.031, NiPc^SO3^/PAH: 0.158 and CuPc^SO3^/PAH: 0.189). Taking into account that the molar extinction coefficients of the three phthalocyanines are of the same order of magnitude, it could be concluded that the CuPc^SO3^/PAH films were more closely packed than NiPc^SO3^/PAH films or FePc^SO3^/PAH films. The preparation method was highly reproducible and coefficients of variation calculated from the maximum absorbance of 20 bilayer films, were lower than 2%.

### 3.2. EQCM Measurements in Glucose and Catechol

In a first approach and in order to test the sensing performance of the EQCM LbL films, they were immersed in catechol (an antioxidant usually found in grape juices) and glucose, one of the major sugars. Cyclic voltammograms (potential range from −1.0 to +1.0 V *vs*. Ag|AgCl) and massograms were recorded simultaneously.

**Figure 2:**
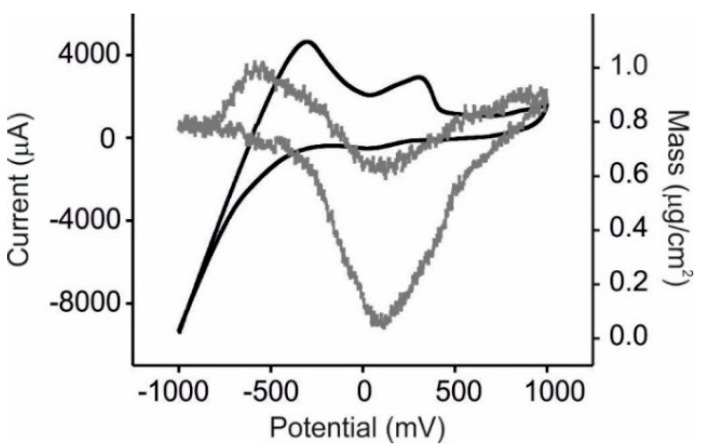
Response of the array of sensors towards catechol 10^−3^ mol·L^−1^ in KCl 0.1 mol·L^−1^. Voltammetric output (black line) and mass output (grey line) for the NiPc^SO3^/PAH sensor.

The responses towards catechol are illustrated in Figure 2 for NiPc^SO3^/PAH films. It is important to remark that in all cases, the first scan was always different from the subsequent cycles. After the second cycle, scans were highly reproducible with a coefficient of variation (%CV) of the highest peak were lower than 2%.

The voltammetric responses were characterized by two redox process, one corresponding to the oxidation/reduction of catechol (at +0.30 V and +0.05 V for the anodic and cathodic waves respectively). The decomposition of water occurring at negative potentials was accompanied by the oxidation of hydrogen that was observed as an anodic wave at −0.45 V. The four electrodes forming the array showed similar trends but the peak positions and their intensities differ from one electrode to another. For instance, the oxidation of catechol occurs at +0.25 V for FePc^SO3^/PAH, +0.30 V for NiPc^SO3^/PAH and +0.42 V for CuPc^SO3^/PAH sensor, indicating that the electrocatalytic effect of the phthalocyanine decreased when advancing in the transition metal series. The intensity of the peaks increased with respect to the values observed in the bare Pt electrode (2780 µA), FePc^SO3^/PAH (2800 µA), NiPc^SO3^/PAH (2930 µA) and CuPc^SO3^/PAH (3260 µA).

Hydrogen was formed during the water decomposition that occurred at ca. −0.45 V according to the next reaction:

(2) $$2H_{2}O+2e^{-}\to H_{2}+2OH^{-}$$

Also in this case, the peak position shifted to more negative values when advancing in the transition metal series (−0.25 V for FePc^SO3^/PAH, −0.35 V for NiPc^SO3^/PAH and −0.42 for CuPc^SO3^/PAH sensor and −0.45 for the Pt bare electrode), confirming the electrocatalytic effect of the nanostructured films.

Massograms showed a mass increase during the oxidation of catechol and a large decrease in mass during the reduction. Massograms were highly stable, however, a net increase in the mass at the end of the experiment was observed, indicating that catechol was adhering to the surface of the electrode. This result is in good accordance with previously published results that demonstrated the passivation of the electrodes during cycling due to polymerization of phenols [58]. In fact, when sensors used to analyze catechol were immersed in a KCl 0.1 mol·L^−1^ solution, the presence of catechol was still observed. According to this, sensors were considered as single use devices and a brand new sensor must be prepared to measure each sample.

**Figure 3:**
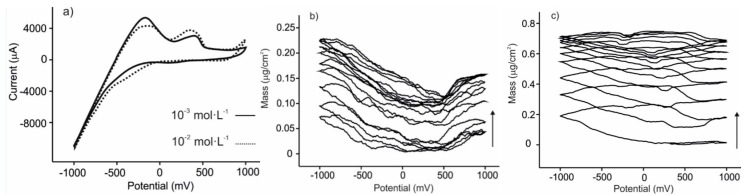
(**a**) Voltammetric response towards catechol in KCl 0.1 mol·L^−1^ for the CuPc^SO3^/PAH sensor; (**b**) Mass response of the CuPc^SO3^/PAH sensor towards glucose 10^−3^ mol·L^−1^ in 0.01 mol·L^−1^ phosphate buffer (pH 7.0); (**c**) Mass response of the CuPc^SO3^/PAH sensor towards glucose 10^−2^ mol·L^−1^ in 0.01 mol·L^−1^ phosphate buffer (pH 7.0).

As expected, the glucose solution did not produce redox peaks in the studied range (except peaks associated to protons at negative potentials). A progressive increase in mass was observed under consecutive cycling that could be attributed to the adhesion of sugars to the sensing surface. The effect of the concentration in the responses of the sensors was analyzed by exposing the EQCM devices to different catechol and glucose concentrations. The results are illustrated in Figure 3 for CuPc^SO3^/PAH. When increasing the concentration of catechol, the voltammetric peak at −0.4 V decreased in intensity, while peak at ca. +0.4 increased in intensity and simultaneously shifts to lower potentials. Similarly, the shape of massograms was different depending on the concentration. This behavior impedes the construction of calibration curves and justifies the need of an array of electrodes, where the whole curve provides information about the sample.

### 3.3. Analysis of Grape Juices

**Figure 4:**
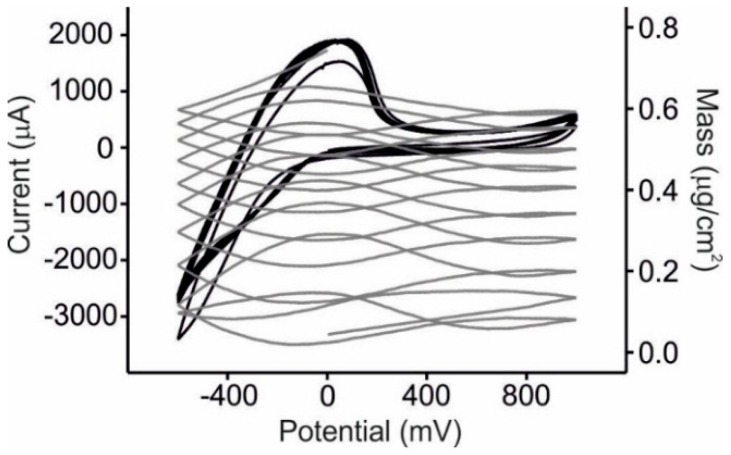
Voltammetric (black line) and mass (grey line) response of the NiPc^SO3^/PAH sensor towards a must obtained from Juan García grapes.

The array of sensors was immersed in must samples prepared from different varieties of grape and EQCM analysis was performed. It has to be pointed out that preliminary experiments were carried out in undiluted must and the signals demonstrated a poor reproducibility. This can be attributed to the large amount of suspended materials (*i.e*. proteins) present in must samples that can adhere to the sensor surface. The reproducibility was clearly improved when musts were diluted 1:2 in KCl 0.05 mol·L^−1^. For this reason, further experiments were carried out in diluted musts. Cyclic voltammetry was carried out at a potential range from −0.6 to +1.0 V *vs*. Ag|AgCl and voltammograms/massograms were simultaneously registered. As an example of the responses obtained Figure 4 shows the voltammetric and mass outputs of NiPc^SO3^/PAH sensor towards a must obtained from the Juan García grape variety.

As usual, the first cycle was different from the rest, but subsequent responses were stable and highly reproducible with coefficients of variation (%CV) within the range of 0.4%–2.8%. Voltammograms were dominated by a broad anodic peak that could be associated to the polyphenolic content of wines [8,59]. On the other hand, the massograms showed a progressive increase in mass. In a complex mixture such as must, it is difficult to establish the process by which the mass increases. Taken into account the results obtained in the presence of catechol and glucose, it can be assumed that polymerization of phenols can play a key role in the observed mass changes. In addition, the adhesion of other main components of musts without redox activity in the studied range such as sugars cannot be neglected.

**Figure 5:**
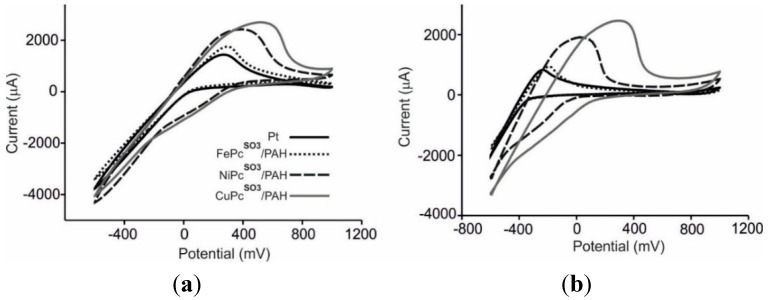
Voltammetric outputs of the EQCM sensors immersed in (**a**) Mencía Regadío; (**b**) Juan García grapes.

The results obtained were similar for all the phthalocyanines tested, but the peak positions and their intensities depend on the phthalocyanine used to form the LbL film. Figure 5 illustrates the voltammetric response of the array of sensors towards musts. The responses obtained towards the Mencía Regadío and the Juan García varieties have been chosen as examples. In good accordance with the results obtained in catechol, the peak positions shifted to higher values when advancing in the transition metal series, confirming that these anodic peaks are associated to the polyphenolic content of grapes.

Because the polyphenolic content varied from one must to another (see Table 1), the positions, broadness and intensities of the peaks were different.

**Figure 6:**
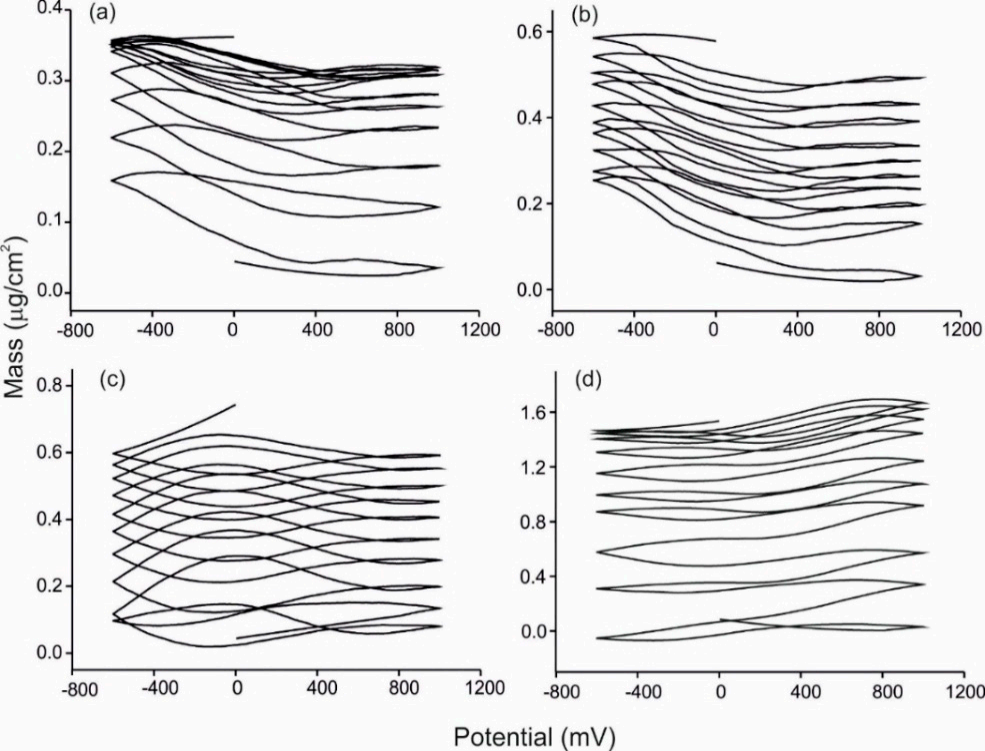
Mass outputs of (**a**) Pt bare sensor immersed in Prieto Picudo; (**b**) FePc^SO3^/PAH sensor immersed in Tempranillo; (**c**) NiPc^SO3^/PAH sensor immersed in Juan García; (**d**) CuPc^SO3^/PAH sensor immersed in Cabernet.

The massograms registered simultaneously to the cyclic voltammetry are illustrated in Figure 6 for different sensors/musts (due to the complexity of the signals it is not possible to superimpose signals for comparison purposes). As expected, all massograms showed a progressive increase in mass, but also in this case, each must produce a different signature. The change in mass could be attributed to the adhesion of sugars and/or to other complex processes and polymerizations. It has to be noticed that in some cases, the adhesion of suspended material caused spikes or a sudden increase in mass. In those cases (15% of the measures), the results were discarded. In the absence of such processes, the experiments were highly reproducible.

### 3.4. Statistical Analysis

The intrinsic complexity, richness and cross-selectivity of the signals generated by voltammetric sensor arrays are an advantage because the dataset contains meaningful information about the sample. As the array of EQCM sensors showed a characteristic voltammetric and mass response depending on the must analyzed, these data could be used to discriminate musts according to their chemical nature. In order to evaluate the capability of discrimination of the system, a non-supervised multivariate technique, the multi-way decomposition PARAFAC method was used.

Prior to statistical analysis, voltammograms and massograms were preprocessed by means of a data reduction technique using “kernels” to obtain 10 variables from each voltammogram and 10 variables from each massogram. The input array for computing the PARAFAC model was a three-way data matrix (“6 must samples with 3 replicas” × “10 kernels” × “4 sensors”), therefore the size was (18 × 10 × 4).

**Figure 7:**
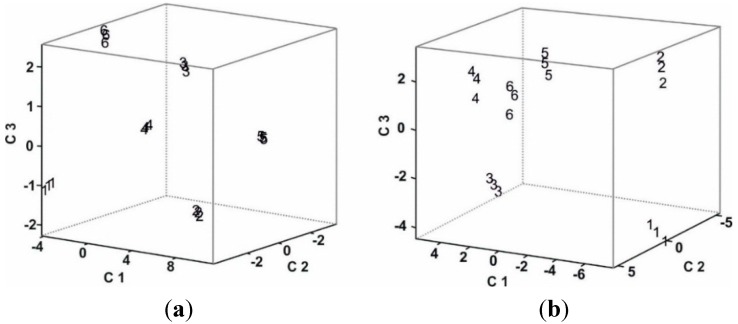
PARAFAC scores plot of the array obtained from (**a**) The voltammetric responses; (**b**) The mass responses. Must samples are 1: Prieto Picudo; 2: Garnacha; 3: Cabernet Saugvinon; 4: Tempranillo; 5: Juan García; and 6: Mencía Regadío.

Figure 7 represents the tridimensional PARAFAC scores plot for the multisensor system using voltammetric outputs. As observed in the figure, the clusters corresponding to the six studied musts (three replicas per must) were clearly separated and were located according to the Total Polyphenol Index (TPI): musts with high TPI (Mencía Regadío, Cabernet Sauvignon, Tempranillo and Juan García variety) appeared in the region corresponding to a positive C3, while musts with low TPI (Prieto Picudo and Garnacha variety) appeared in the negative C3 values region. This good discrimination can be explained taking into account that voltammograms reflect the redox activity of the phenols. The error in terms of root-mean-square error (RMSE) was 0.4551.

Figure 7 also shows the tridimensional PARAFAC scores plot when analyzing the mass output. In this case RMSE was 0.6472. Massograms also allowed a good-quality discrimination. However, the positions of the clusters were not related to the Total Polyphenol Index. Instead, their positions were located according to the sugar content.

Musts with the highest Brix degree (Cabernet Sauvignon and Prieto Picudo) were located at negative values of C3, whereas those with lower sugar content at positive values of C3 (Tempranillo, Mencía Regadío, Juan García and Garnacha). This result confirms that changes in mass observed from one massogram to the subsequent one are due to the adhesion of sugars.

Prediction models were established by N-PLS. The classification models were subjected to full cross-validation by means of the “leave-one-out” method. N-PLS was carried out to establish correlations between the EQCM signals obtained from the array and chemical parameters. N-PLS regression builds a calibration model incorporating a relationship between the sets of predictors and responses based on the multiway structure of the arrays. Calibration fits the model to the available data, while validation checks the model for new data. Results are shown in Table 2 and Table 3.

Correlations were obtained with the voltammetric data, which showed higher coefficients of correlations and lower errors. Results indicate that the voltammetric signals are better correlated with the TPI with only three components (Table 2).

The mass outputs present a better correlation with those parameters related to the sugar content (sugar content, degree 16.8 and Brix degrees) (Table 3) than with those related to the polyphenolic content. The correlations calculated between mass data and chemical parameters showed lower correlation coefficients and higher errors than the correlations obtained with voltammetric data.

**Table 2:** Statistical parameters obtained for the N-PLS regression model established between the chemical parameters and the voltammetric responses of the sensors towards musts.

Voltammetric Outputs					
Parameters	R^2^_C_^(a)^	RMSEC ^(b)^	R^2^_P_^(c)^	RMSEP ^(d)^	Number of Components
Sugar content	0.997	0.99187	0.945	4.24917	4
Brix degree	0.996	0.09242	0.935	0.40019	4
Degree 16.8	0.997	0.05894	0.946	0.25147	4
TPI	0.992	0.46538	0.983	0.68089	3
Polyphenolic content	0.998	0.33442	0.989	1.11841	3
Folin-Ciocalteau method					

^(a)^ Squared correlation coefficient in calibration; ^(b)^ Root Mean Square Error of Calibration; ^(c)^ Squared correlation coefficient in prediction; ^(d)^ Root Mean Square Error of Prediction.

**Table 3:** Statistical parameters obtained for the N-PLS regression model established between the chemical parameters and the mass responses of the sensors towards musts.

Mass Outputs					
Parameters	R^2^_C_^(a)^	RMSEC ^(b)^	R^2^_P_^(c)^	RMSEP ^(d)^	Number of Components
Sugar content	0.941	4.45005	0.839	7.31293	4
Brix degree	0.972	0.00176	0.865	0.00291	4
Degree 16.8	0.941	0.26438	0.840	0.43420	4
TPI	0.961	1.02511	0.845	2.04940	5
Polyphenolic content	0.965	1.91428	0.921	3.0353	5
Folin-Ciocalteau method					

^(a)^ Squared correlation coefficient in calibration; ^(b)^ Root Mean Square Error of Calibration; ^(c)^ Squared correlation coefficient in prediction; ^(d)^ Root Mean Square Error of Prediction.

As voltammetric and mass data are registered simultaneously, EQCM can improve the discrimination of must samples while providing good correlations with both sugar and polyhenolic content.

## 4. Conclusions

A multisensor system formed by EQCM sensors modified with LbL films containing phthalocyanines have been successfully fabricated and used to discriminate musts obtained from different varieties of grapes. The capability of the sensor array to discriminate grapes according to their chemical parameters has been evidenced using the multivariate decomposition method PARAFAC. Voltammograms showed anodic peaks that were related to the phenolic content in musts and massograms showed an increase of mass that was related to the sugar content of the musts. Prediction models performed by multi-way partial least squares (N-PLS) have showed correlations between the voltammetric and mass outputs with the chemical parameters. EQCM has demonstrated to be advantageous because voltammetric and mass data can be registered simultaneously, providing good correlations with the sugar and the polyhenolic content, which are the most important indicators of quality and degree of ripeness in grapes.
